# Deep learning enabled ultra-high quality NMR chemical shift resolved spectra†

**DOI:** 10.1039/d4sc04742g

**Published:** 2024-11-11

**Authors:** Zhengxian Yang, Weigang Cai, Wen Zhu, Xiaoxu Zheng, Xiaoqi Shi, Mengjie Qiu, Zhong Chen, Maili Liu, Yanqin Lin

**Affiliations:** a Department of Electronic Science, Fujian Provincial Key Laboratory of Plasma and Magnetic Resonance, State Key Laboratory of Physical Chemistry of Solid Surfaces, Xiamen University Xiamen Fujian 361005 China linyq@xmu.edu.cn; b State Key Laboratory of Magnetic Resonance and Atomic and Molecular Physics, National Center for Magnetic Resonance in Wuhan, Wuhan Institute of Physics and Mathematics, Innovation Academy for Precision Measurement Science and Technology, Chinese Academy of Sciences Wuhan 430071 China; c University of Chinese Academy of Sciences Beijing 100049 China

## Abstract

High quality chemical shift resolved spectra have long been pursued in nuclear magnetic resonance (NMR). In order to obtain chemical shift information with high resolution and sensitivity, a neural network named spin echo to obtain chemical shifts network (SE2CSNet) is developed to process the NMR data acquired by the spin echo pulse sequence. Through detecting the change of phase in the spin echo spectra, SE2CSNet can accurately detect the chemical shift position of spectral signals. The results show that the network can discern the chemical shift even when spectral signals overlap, but without strong coupling and chunking artifacts. In addition, this method can process the sample with low S/N (signal to noise ratio), and recover weak signals even hidden in noise, leading to ultra-high quality chemical shift resolved spectra. It is envisioned that the proposed methodology will find wide applications in many fields.

## Abstract 2

Accurate chemical shifts of spectral signals were obtained by processing spin echo spectra using a deep learning method.

## Graphical abstract

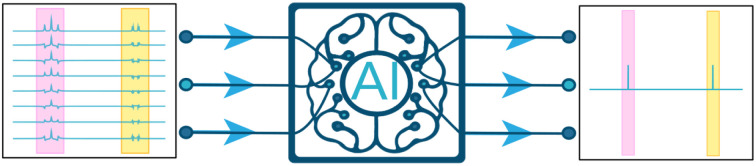

## Introduction

^1^H nuclear magnetic resonance (NMR) is an extensively utilized analytical technique that can be used for structural analysis of compounds and even proteins with complex structures.^1^ However, multiplet splitting caused by scalar coupling (*J*-coupling) plus the narrow chemical shift range often leads to severe spectral overlapping and low spectral resolution,^2^ hampering correct identification of chemical shifts of spectral signals. Addressing this defect is crucial for effective spectral analysis.

Pure shift techniques convert multiplets into singlets by removing *J*-coupling, simplifying spectral information and significantly improving signal dispersion and thus spectral resolution. The Pure Shift Yielded by Chirp Excitation (PSYCHE)^3^ method based on pseudo 2D sampling and the real-time Zangger-Sterk (ZS)^4^ method based on real time sampling are the representative pure shift techniques, proposed in 2014 and 2013 respectively. Both PSYCHE and real-time ZS methods suffer from severe sensitivity loss and artifact interference,^5^ resulting in a significant decrease in the spectral quality and thus limited practical applications. The emergence of ultra-high-field NMR spectrometers, such as the 1.2 GHz one,^6,7^ can partially overcome the issue, but with a large economic cost.

In recent years, deep learning (DL) methods have gained widespread adoption across various fields, including NMR non-uniform sampling reconstruction, denoising, spectral assignment and structural analysis, and improving spectral quality. In the field of NMR non-uniform sampling reconstruction, several innovative approaches have been developed. In 2019, Hansen *et al.* introduced a novel network architecture based on Long Short-Term Memory (LSTM) layers to reconstruct sparsely sampled NMR spectra in the time domain,^8^ while Qu *et al.* demonstrated the application of deep neural networks for achieving high-quality and reliable reconstructions in the frequency domain.^9^ In 2020, Luo *et al.* proposed a deep neural network named EDHRN, designed for the fast reconstruction of non-uniformly sampled multidimensional NMR spectroscopy.^10^ More recently, in 2022, Zheng *et al.* presented a deep learning-based method to accelerate the acquisition of undersampled PSYCHE spectra.^11^ In the field of NMR denoising, Wu *et al.* proposed a deep neural network named DN-Unet in 2020, specifically designed to suppress noise in liquid-state NMR spectra.^12^ In the field of spectral assignment and structural analysis, several notable advancements have also been made. In 2021, Li *et al.* introduced DEEP Picker, a deep neural network (DNN)-based approach for peak picking and spectral deconvolution, which semi-automates the analysis of two-dimensional NMR spectra.^13^ In 2022, Klukowski *et al.* presented ARTINA, a deep learning-based method that delivers signal positions, resonance assignments, and structures without human intervention, using only NMR spectra and the protein sequence as input.^14^ Building on this, in 2023, they introduced an integrative approach combining ARTINA with AlphaFold and UCBShift, enabling chemical shift assignment with reduced experimental data and increased accuracy.^15^ And in the field of improving spectral quality, in 2023, Xiao *et al.* proposed a data postprocessing method, which uses a convolutional neural network named RH-Unet to restore high quality spectra from distorted ones that were acquired in inhomogeneous magnetic fields.^16^ Additionally, Yang *et al.* developed a neural network named AC-ResNet and a loss function named SM-CDMANE to obtain high-quality NMR spectra from low-quality pure shift NMR data based on real-time ZS.^17^

In this study, a new neural network named spin echo to obtain chemical shifts network (SE2CSNet) is proposed to process several spin echo spectra acquired with different echo times, and obtain ultra-high resolution chemical shift resolved ^1^H NMR spectra without artifact interference. This method has the same, or even higher sensitivity compared to conventional 1D pulse sequences, and thus can be used to analyze low concentration samples, greatly outperforming current popular pure shift techniques. During the preparation of the paper, a similar idea^18^ appears on a preprint server.

## Methods and materials

**Fig. 1:**
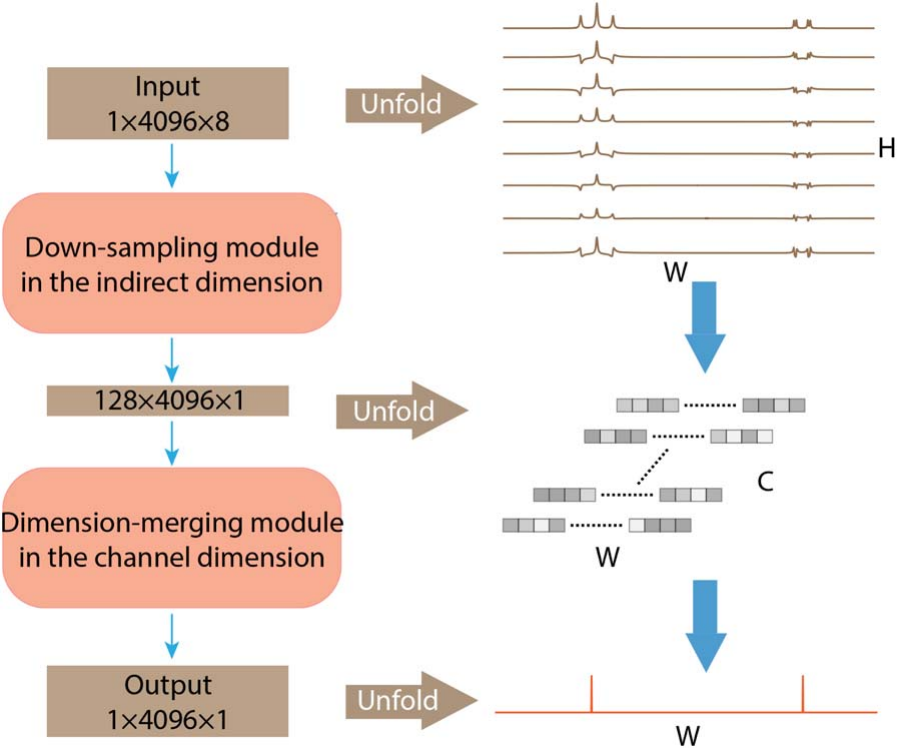
The architecture of SE2CSNet. The data size is represented by *C* × *W* × *H*, where *W* is the size in the direct dimension or the width of the feature matrix, *H* is the size in the indirect dimension or the height of the feature matrix, and *C* is the number of channels.

The architecture of SE2CSNet is shown in Fig. 1. The network is based on the convolutional neural network and uses the residual block structure of widely used ResNet.^19^ 8 Spin echo phase-changed spectra with different echo times are input into SE2CSNet to obtain the chemical shift matrix. SE2CSNet features down-sampling in the indirect dimension (*H* in Fig. 1) and dimension-merging in the channel dimension (*C* in Fig. 1). Through the down-sampling module, the input data with the size of H in the indirect dimension are fused to correlate the input eight spectra with each other, obtaining the data with the size of one in the indirect dimension, and channels are expanded from one to *C* to better preserve feature information. Through the dimension-merging module, the feature vector with the size of *C* is merged together into one in the channel dimension to extract effective information, getting the desired one-dimensional chemical shift resolved spectrum. In this module, merging is performed in a step-by-step manner (Fig. S1b†) to reduce the channel size, which alleviates the problem of misidentifying signal chemical shifts and thus improves the accuracy of the network model, and a SoftMax activation function for making the feature sparser is employed in each step, as shown in Fig. S1b.† This approach produces better results compared with that from typical network design with merging in one step and employing only one SoftMax activation function, as shown in Fig. S3.†

The dataset of spin echo phase-changed spectra was simulated to train SE2CSNet, consisting of 40 000 samples. The input dataset can be obtained based on the spin evolution of the spin echo pulse sequence, which is shown in Fig. S4.† In the spin echo pulse sequence, the spin evolution formula of one signal can be expressed as:1
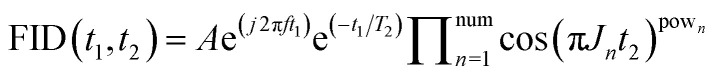
where *A*, *j*, *f*, *T*_2_, num, *J*, and pow respectively represent amplitude, imaginary unit, frequency, transverse relaxation time, the number of ^1^H groups, *J*-coupling constant and the number of coupled spins. The ranges of these parameters are shown in Table S1.†*t*_1_ is the evolution time of chemical shift, and *t*_2_ is the evolution time of transverse relaxation and *J*-coupling. The range of *t*_1_ is 0 ∼ (np − 1)/SW, and the range of *t*_2_ is 2*τ* ∼ 2*τ* + (np − 1)/SW, where SW is spectral width, np is the number of sampling points (fixed at 4096), and 2*τ* is the echo time.

The signals of different spins in a spin echo spectrum are modulated by different *J*-couplings, resulting in different phases. Additionally, the same signals in different spin echo spectra are influenced by different echo times, leading to distinct phases as well. Therefore, SE2CSNet can distinguish and detect the signals of different spins based on the features of the changed phases.

The label data is a one-dimensional signal matrix with 4096 points (the same length of the spin echo spectrum). In the signal matrix, the central positions of the signals are set to 1 with their values as chemical shift, and other positions are set to 0. The labels not only provide a confidence score but also indicate the presence of a signal. A confidence score of 1 suggests that there is a 100% certainty of a signal with this specific chemical shift, while a score of 0 indicates that there is a 0% certainty signal.

Mean Absolute Error (MAE) was used as the loss function during the training process, and it is denoted as:2
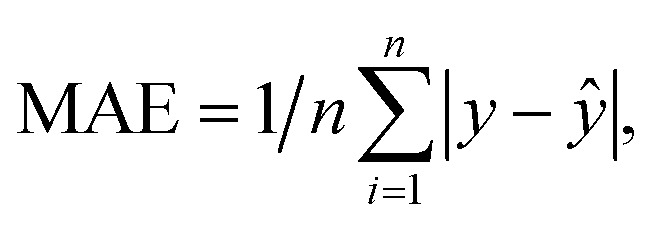
where *y* and *ŷ* are the output of the model and the label, respectively. The network outputs the confidence of each point after training, and a decision threshold is applied to discard low-confidence predictions, resulting in the final binary spectrum (0 or 1). In this study, a decision threshold of 0.5 (the F1 score is relatively high at this value) is used with how to determine the threshold shown in the ESI,† meaning that a confidence score of 0.5 or higher indicates that the prediction of the signal at that point is considered reliable, while a score below 0.5 indicates that the prediction is not.

The structural details and training details of SE2CSNet and more details of the simulated dataset are provided in the ESI.†

To evaluate the performance of the trained SE2CSNet on signal identification, 10 000 sets of simulated data were generated to compute the precision and recall. Precision is the ratio of the number of true positives predicted by the network to the total number of positives predicted by the network, as shown by *P* of eqn (3). *P*, TP and FP are precision, the number of true positives and the number of false positives, respectively.

3 *P* = TP/(TP + FP),

Recall is the ratio of the number of true positives predicted by the network to the total number of actual positives, as shown by *R* of eqn (4). *R*, TP and FN are recall, the number of true positives and the number of false negatives, respectively.

4 *R* = TP/(TP + FN),

The F1 score is the harmonic mean of precision and recall and offers a more comprehensive performance evaluation, as shown by F1 of eqn (5). F1, *P* and *R* are the F1 score, precision and recall, respectively.

5 F1 = 2 × (*P* × *R*)/(*P* + *R*),

In the 10 000 simulated datasets, there are a total of 295 607 signals, including 2225 false positives and 278 094 true positives. The calculated precision is 99.21%, while the recall is 94.08%, resulting in an F1 score of 0.93. The high precision indicates that SE2CSNet is highly reliable in predicting positive signals, with very few false positives. However, although the recall is slightly lower than the precision, it still demonstrates that most signals are correctly identified, with only a few being missed. This discrepancy could be attributed to the variability or complexity of signal features in the simulated data. Nonetheless, the high F1 score demonstrates that SE2CSNet achieves a good balance between precision and recall, thereby showing overall effective performance in signal detection.

**Fig. 2:**
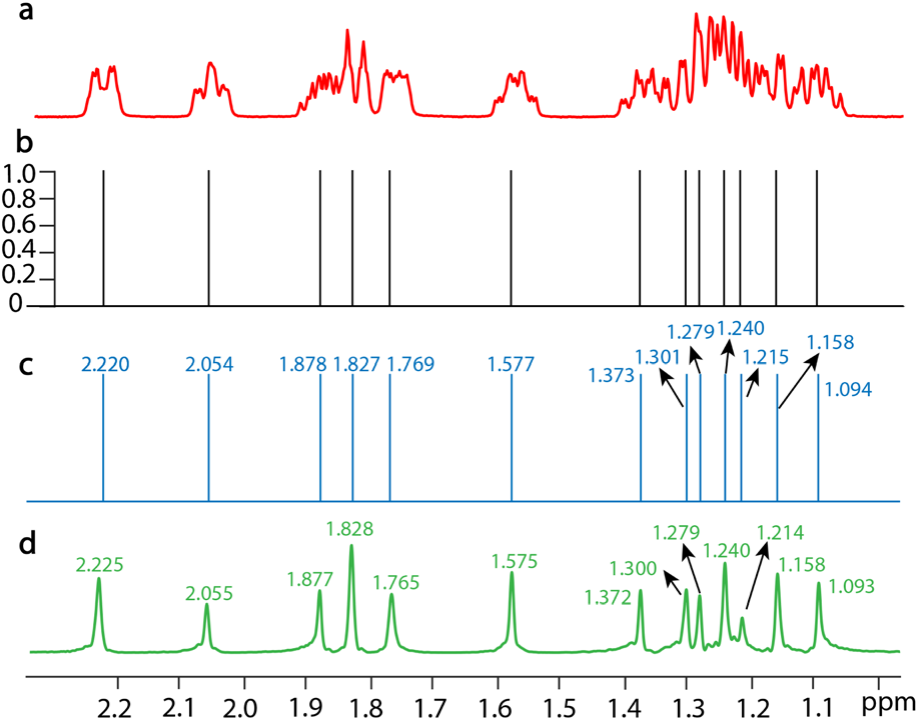
1D NMR spectra of 50 mM estradiol dissolved in (methyl sulfoxide)-d6 (DMSO-d6) with a scan number of one. (a) The spin echo spectrum with an echo time of 0 s. (b) The confidence result produced by SE2CSNet. (c) The output chemical shift spectra produced by SE2CSNet. (d) The PSYCHE spectrum as a reference.

**Fig. 3:**
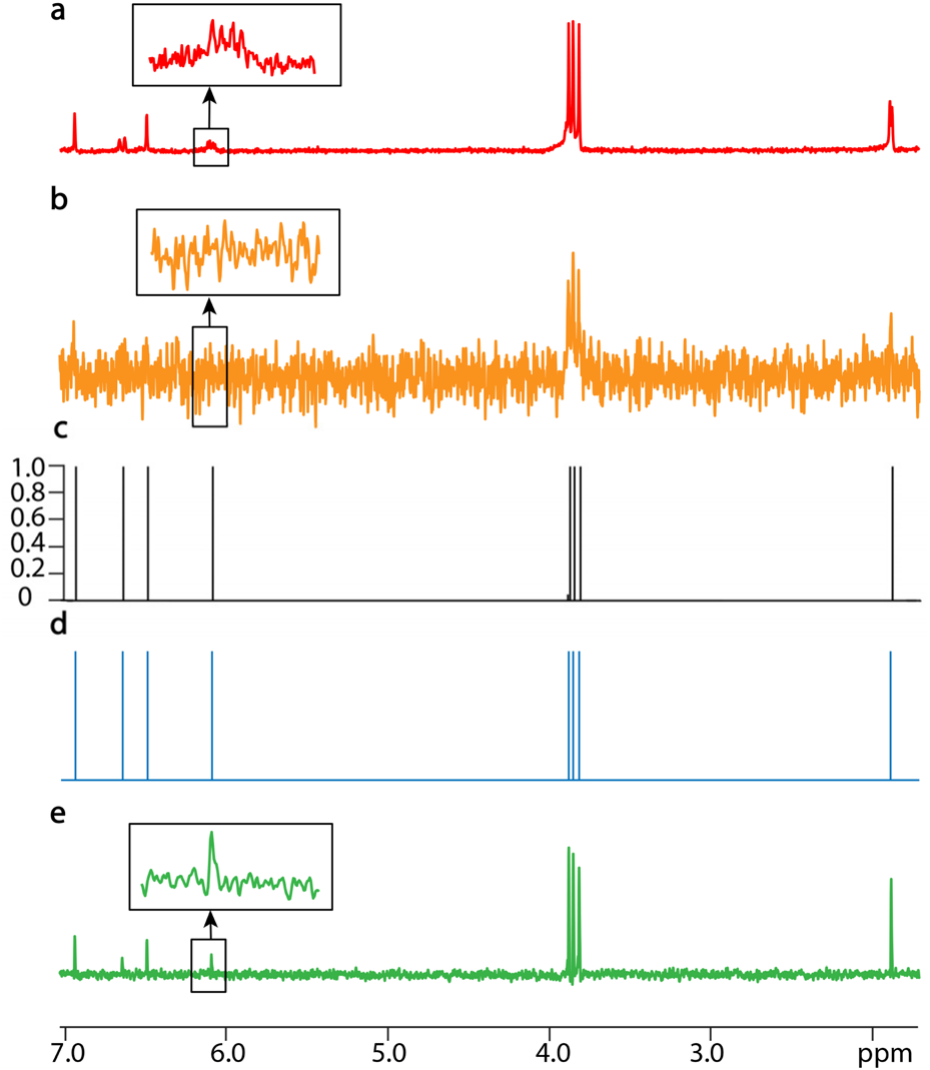
1D NMR spectra of 1 mM (a, b, and d) and 30 mM α-asarone (e) dissolved in chloroform-d (CDCl_3_) with a scan number of one. (a) The spin echo spectrum with an echo time of 0 s. (b) The PSYCHE spectrum. (c) The confidence result produced by SE2CSNet. (d) The output chemical shift spectra produced by SE2CSNet. (e) The PSYCHE spectrum as a reference. Representative regions are expanded.

**Fig. 4:**
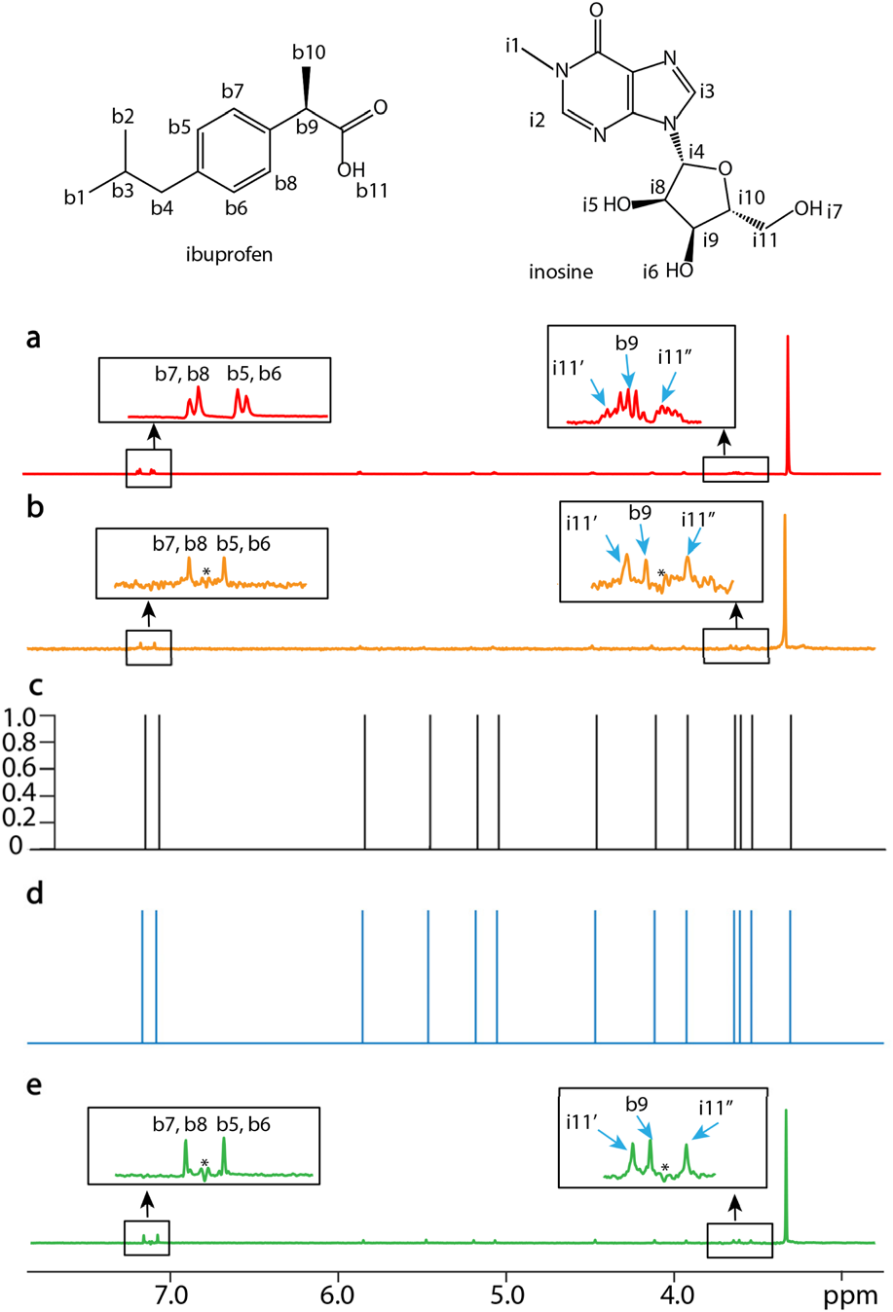
1D NMR spectra of the mixture of ibuprofen and inosine dissolved in (methyl sulfoxide)-d6 (DMSO-d6) with a scan number of one. (a) The spin echo spectrum of 3 mM ibuprofen and 2 mM inosine with an echo time of 0 s. (b) The PSYCHE spectrum with the same sample as (a). (c) The confidence result produced by SE2CSNet. (d) The output chemical shift spectra produced by SE2CSNet. (e) The PSYCHE spectrum of the mixture with 9 mM ibuprofen and 6 mM inosine. Representative regions are expanded. Blue arrows indicate representative signals and ‘*’ indicates strong coupling artifacts.

**Fig. 5:**
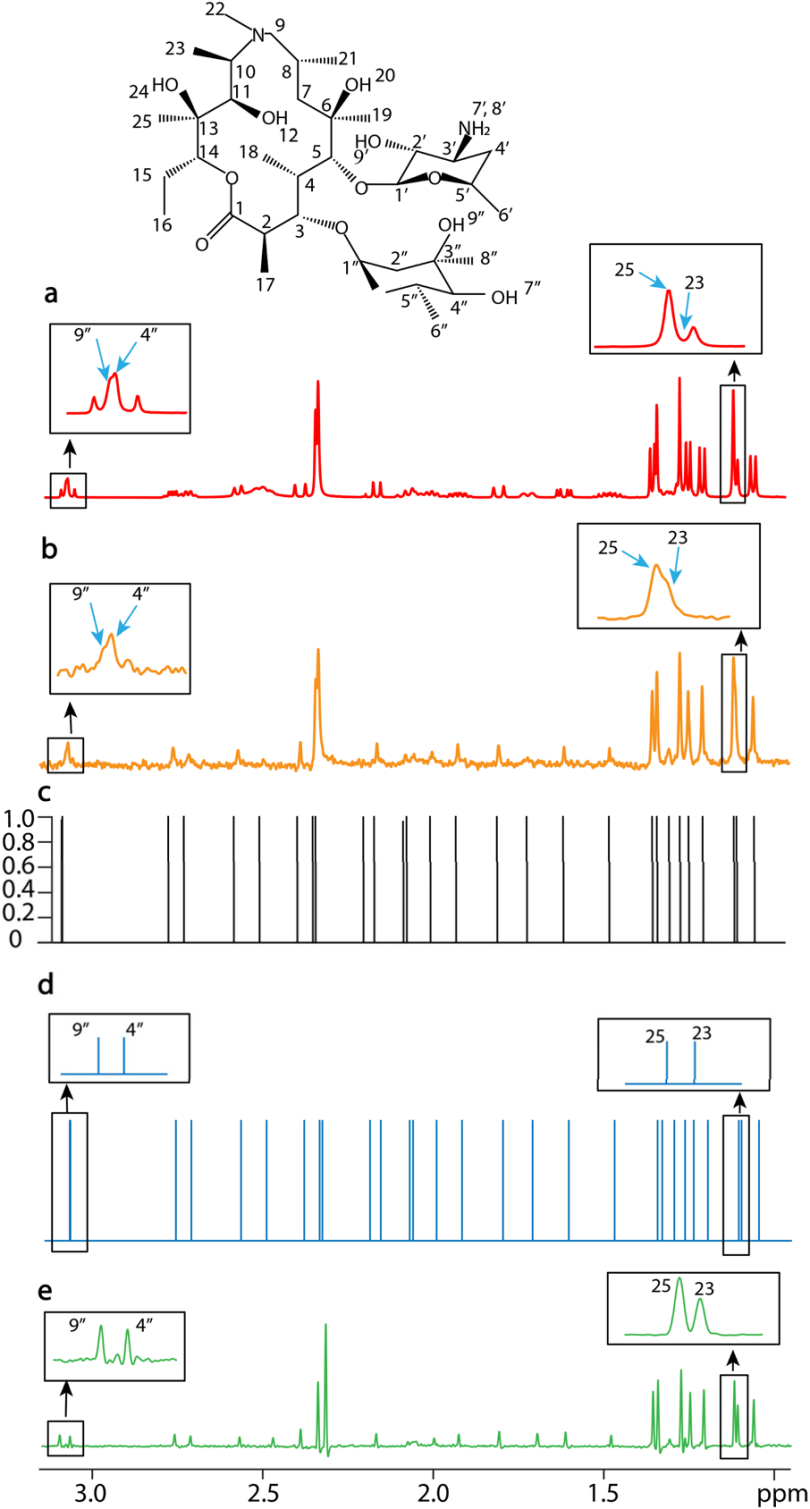
1D NMR spectra of 20 mM (a, b, and d) and 40 mM azithromycin (e) dissolved in chloroform-d (CDCl_3_) with a scan number of one. (a) The spin echo spectrum with an echo time of 0 s. (b) The PSYCHE spectrum. (c) The confidence result produced by SE2CSNet. (d) The output chemical shift spectra produced by SE2CSNet. (e) The PSYCHE spectrum as a reference. Representative regions are expanded. Blue arrows indicate representative signals.

In order to demonstrate the performance of SE2CSNet on actual samples, samples of 50 mM estradiol, 1 mM α-asarone (C_12_H_16_O_3_), the mixture of 3 mM ibuprofen (C_13_H_18_O_2_) and 2 mM inosine (C_10_H_12_N_4_O_5_), and 20 mM azithromycin (C_38_H_72_N_2_O_12_) are used, and the results are shown in Fig. 2–5 respectively. All experimental data were acquired on a 500 MHz Varian NMR spectrometer at 298 K. The FID undergoes manual baseline correction and phase correction in VnmrJ software. To match the input dimensions for the network, the FID is zero-filled to 4096 points or an integer multiple of 4096 (see the ESI† for a detailed description of zero-filling).

## Results and discussion

SE2CSNet can accurately identify the chemical shift positions of signals. 8 spin echo spectra of 50 mM estradiol (C_18_H_24_O_2_) are shown in Fig. S5† and the processed results are shown in Fig. 2, and chemical shifts of all the signals are marked in the corresponding spectra (Fig. 2b and c). By comparing chemical shift values in the processed spectrum with those in the PSYCHE spectrum as a reference, it can be found that the difference in chemical shift values is always less than 0.005 ppm (it is noted that the difference is very small and thus three significant figures are used). This shows that SE2CSNet can correctly identify chemical shift positions with very high precision.

SE2CSNet can discern signals from strong noise, thus enabling analysis of spectra from low concentration samples. The processed results of 1 mM α-asarone (C_12_H_16_O_3_) are shown in Fig. 3. It is worth noting that the signals in the 2 to 7 ppm range are relatively weak. To better display these signals in the spectrum, both the signal and noise were amplified to a greater extent during the plotting process compared to other samples, which results in a visually enhanced perception of noise. With the PSYCHE spectrum of 30 mM α-asarone as a reference (Fig. 3d), in the PSYCHE spectrum of 1 mM α-asarone (Fig. 3b), weak signals are completely hidden in the noise (see the expanded region), and their spectral information is lost. Even in the spin echo spectrum with the echo time of 0 s (Fig. 3a), equivalent to the spectrum acquired by a conventional 1D single pulse, the signal in the expanded region is heavily disturbed by noise. SE2CSNet can discern these weak signals from strong noise and obtain correct chemical shift values of all signals. Moreover, as shown in Fig. S6,† after adding additional random noise into the original spin echo spectrum, these weak signals are completely overwhelmed in the noise, but SE2CSNet can still correctly discern them. This shows that SE2CSNet has better sensitivity than the conventional 1D pulse sequence, and thus can deal with samples with low concentrations.

SE2CSNet can discern overlapped signals, and thus processes the spectra of relatively complex samples. The processed results of the mixture of 3 mM ibuprofen (C_13_H_18_O_2_) and 2 mM inosine (C_10_H_12_N_4_O_5_) are shown in Fig. 4. In both PSYCHE spectra of the low (Fig. 4b) and high (Fig. 4d) concentration samples, there are strong coupling artifacts (a strong coupling artifact in NMR refers to an incorrect or misleading spectral feature that arises due to strong coupling interactions between nuclear spins, which occurs when the *J*-coupling between two or more nuclear spin systems is comparable to or greater than their chemical shift differences. This phenomenon can complicate the interpretation of NMR spectra, especially in systems where multiple nuclei are closely coupled) indicated by *, causing potential signal misidentification. SE2CSNet is free from the interference of the strong coupling artifacts and correctly identifies the real signal.^20,21^ In the expanded region of the spin echo spectrum in Fig. 4a, two multiplets (signal-i11′ and signal-b9) are partially overlapped. SE2CSNet still has the ability of correct identification of overlapped multiplets.

Azithromycin (C_38_H_72_N_2_O_12_) was used to test the capability of SE2CSNet to process complex samples, as shown in Fig. 5. The spectrum of azithromycin (Fig. 5a) has many signals, and severe signal overlapping prevents correct identification of signals (see expanded regions in Fig. 5a). Even in the PSYCHE spectrum of the same sample (Fig. 5b), signals are still overlapped (see expanded regions in Fig. 5b, signal-9′′, signal-4′′ and signal-23, signal-25), leading to misidentification. SE2CSNet can distinguish these signals by comparing the difference of signal related-phases in the spin echo phase-changed spectrum. Therefore, the network model can correctly identify the chemical shift position of these signals, despite coinciding almost completely. It is noted that the chemical shifts of signal-9′′ and signal-25 vary with concentration (Fig. S7†), and the two overlaps (see the two expanded regions) occur at the concentration of 20 mM.

For multiplets with scalar coupling splitting, their phases do vary with echo time. In contrast, singlets exhibit no apparent phase change. However, this absence of phase change is also recognized by SE2CSNet as a special case, allowing it to identify singlets correctly.

One of the most important tasks in NMR spectral processing is to discern weak signals from strong noise. In order to test the limit of SE2CSNet to discern weak signals from noise, the weakest signal-to-noise ratio (wS/N) is introduced, which is defined as the height of the weakest signal in the spectrum divided by the standard deviation of the noise region with no signal. The testing result is shown in Fig. S8.† The result shows that when wS/N is greater than 4, the network can stably discern weak signals from noise.

In addition, it is also very important to accurately identify the signal in the spectral region with severe signal overlapping for NMR spectral processing. SE2CSNet can correctly distinguish the chemical shifts of overlapping signals by detecting the phase variation even if overlaps are severe, as shown in Fig. S9† (one case is two doublets with slightly different chemical shifts and the other is a singlet having the same chemical shift as that of the splitting signals of a doublet). These results show that the network has an excellent ability to identify overlapping signals with very high accuracy.

SE2CSNet was trained on spectra with only weak couplings. However, the phase distortions were introduced in the simulated spectra of the training dataset, making spectra resemble those with strong couplings, and thus the network has a certain ability to process spectra with strong coupling. To verify this, simulated strong coupling signals with different *α* were generated, where *α* is the factor that accounts for roofing effects (referred to as the strong coupling factor, used to control the degree of strong coupling). The evaluation results reveal that when *α* is not larger than 0.74, SE2CSNet can correctly identify the signals, as shown in Fig. S11.†

Besides, the network was also tested under other adverse conditions including large one-bond *J*-coupling constants, phase distortion, large line widths, and strong solvent signals. For large one-bond *J*-coupling constants, the network can accommodate *J*-coupling constants up to 24 Hz (Fig. S10†). As for phase distortion, the critical values of *r* (the parameter that determines the degree of phase distortion) are −1.59 radians and 1.4 radians (Fig. S12†). Regarding large line widths, the maximum line width that the network can accommodate is 22 Hz (Fig. S13†). For strong solvent signals (Fig. S14†), the network can identify the signal when the ratio of the lowest signal to the highest peak is not smaller than 0.1%.

Experimental data was used to evaluate the network's performance under poor shimming and pulse miscalibrations. As for poor shimming, the network was tested on the spectrum of ibuprofen after increasing the value of *Z*_1_ shimming coil by 80 and spectrum after increasing the value of shimming coil *Z*_2_ by 150 compared to optimal shimming. As shown in Fig. S15,† under the two conditions, SE2CSNet still retains the ability to correctly identify the signal. For pulse miscalibrations, the network was tested on the spectrum of ibuprofen which was acquired using 80° and 160° pulses (Fig. S16a†), and using 140° pulses and 280° pulses (Fig. S16b†) replacing 90° and 180° pulses. As shown in Fig. S16,† SE2CSNet still retains the ability to correctly identify the signal under non-optimal pulse flip angles.

It is also one of the objectives of the NMR spectral processing task to obtain spectra free from artifact interference, have a smooth baseline and small line width. The network is free from the interference of strong coupling artifacts, and obtains the chemical shift information of real signals rather than artifacts. The chemical shift resolved spectra formed by these correctly recognized signals are based on vertical lines with no width. Therefore, although there is no signal intensity information, the chemical shift information is of ultrahigh resolution, even higher than PSYCHE.

Saving time and cost is an important pursuit of NMR methods. In this paper, it takes about 1 minute and 40 seconds to obtain the PSYCHE spectrum with one average. It takes about 20 seconds to obtain spin echo spectra with one average, and about 2 seconds to process the spectra. The total time of the proposed method is much shorter than that of the PSYCHE method.

The network outputs confidence values that are mostly close to 1 or close to 0. This is because the network is designed to learn the significant differences between signals and non-signals in the spectra. Specifically, the characteristics such as phase in spin echo phase-changed spectra make it easy to distinguish between signals and non-signals, allowing the network to learn these features and make strong classification decisions. The label design aligns with this goal (the central positions of the signals are set to 1 with their values as chemical shifts, while other positions are set to 0). And thus the network tends to generate “extreme” confidence outputs, with values close to 0 or 1, indicating precise judgments of signals or non-signals.

## Conclusions

In summary, we propose a neural network called SE2CSNet in this study, designed for effectively extracting the positions of spectral signals by leveraging crucial features in the spin echo spectra, *i.e.* phase change. Notably, the acquisition of spin echo phase-changed spectra is straightforward and time-efficient. After passing through the network, the output spectra provide accurate pure chemical shift information. Additionally, even outside the parameter range of the training set, the network still exhibits a certain level of signal identification ability, suggesting the network's good generalization. The versatility of this method allows it to handle diverse sample types, demonstrating immense potential across various fields.

## Code availability

The trained model and code for SE2CSNet are available at https://github.com/LinYanqin/SE2CSNet.

## Data availability

The experimental data of four samples that support the findings of this study are available at https://github.com/LinYanqin/SE2CSNet.

## Author contributions

Y. L., Z. Y. and W. C. conceived the study. Z. Y. and W. C. designed and optimized the network. W. C., Z. Y., X. Z., X. S. and W. Z. conducted the experiments and analyzed the results. Z. Y., W. C. and M. Q. prepared the manuscript. Z. C. and M. L. helped with designing the study and interpreting the data. The manuscript was written through contributions of all authors. All authors have given approval to the final version of the manuscript.

## Conflicts of interest

There are no conflicts to declare.

## Supplementary Material

SC-015-D4SC04742G-s001
