# Supplementary material for: Deep learning enabled ultra-high quality NMR chemical shift resolved spectra
Source: Chem Sci. 2024 Nov 11;15(47):20039–44. doi: 10.1039/d4sc04742g (PMC11575604; doi:10.1039/d4sc04742g)
Supplement: SC-015-D4SC04742G-s001 [file SC-015-D4SC04742G-s001.pdf]

Supplementary Information (SI) for Chemical Science.
This journal is © The Royal Society of Chemistry 2024

Supporting Information

Deep Learning Enabled Ultra-high Quality NMR Chemical Shift Resolved Spectra

Zhengxian Yang,[†a] Weigang Cai,[†a] Wen Zhu,[a] Xiaoxu Zheng,[a] Xiaoqi Shi,[a] Mengjie Qiu,[a] Zhong Chen,[a] Maili Liu,[b] and Yanqin Lin[*a]

[a]Department of Electronic Science, Fujian Provincial Key Laboratory of Plasma and Magnetic Resonance, State Key Laboratory of Physical Chemistry of Solid Surfaces, Xiamen University, Xiamen, Fujian 361005, China

[b]State Key Laboratory of Magnetic Resonance and Atomic and Molecular Physics, National Center for Magnetic Resonance in Wuhan, Wuhan Institute of Physics and Mathematics, Innovation Academy for Precision Measurement Science and Technology, Chinese Academy of Sciences, Wuhan 430071, China; University of Chinese Academy of Sciences, Beijing 100049, China

[†]These authors contributed equally to this work.

[*]Email: linyq@xmu.edu.cn

Table of Contents

1. Supplementary Text

2. Fig. S1 to 16

3. Table S1

Supplementary Text

The structural details of SE2CSNet

The details of the two modules and the residual block are shown in Fig. S1. The data size is represented as 'C × W × H' in the brown matrix, where 'C', 'W' and 'H' denote the number of channel, width and height of the data respectively. And the visual outputs of the two modules are shown in Fig. S2.

Training details of SE2CSNet

The network model was trained on a server with an NVIDIA GeForce GTX 1080 TI GPU. The PyTorch toolkit was used to train and test the network model. The initial learning rate was set to 0.01, and when the validation loss value no longer decreased, the learning rate was reduced to one-tenth of the original. Adam optimization with default values, epoch of 30 and batch size of 50 were used. There are 40000 sets of simulated spin echo phase-changed spectra in the dataset. The total training time was 15 hours.

The input and output of SE2CSNet

The input (the spin echo phase-changed spectrum) and the output (the processed spectrum) of the network model are both 4096 points. FID (free induction decay) containing less than 4096 complex points can be zero-filled to 4096 points. If the FID contains more than 4096 complex points, it can be filled with zero to 8192 or more (integer multiples of 4096). Then the spectrum is divided into two or more spectra, spectra with 4096 points are used as the input of the network, and finally the processed spectra are spliced to get a complete spectrum.

The more details of simulated data

Multi-signals spin echo FID_{1D} (free induction decay) can be obtained by adding *N* (the number of signals) FIDs. Then use Fourier transform to transform FID_{1D} into the spectrum $SPEC_0$, and then normalize $SPEC_0$ and perform inverse Fourier transform to obtain FID_{norm}. Gaussian white noise is then added to the real and imaginary parts of FID_{norm} to obtain FID_{noise}. The range of the noise intensity coefficient is [0, 8×10^{-5}]. Then, Fourier transform FID_{noise} into spectrum $SPEC_{noise0}$, take the real part of $SPEC_{noise0}$ as $SPEC_{noise1}$, and then normalize $SPEC_{noise1}$ as $SPEC_{1D}$. Finally, the simulated input data, i.e. eight spin echo phase-changed spectra (4096×8), are obtained using 8 different echo times (2τ), which are 0, 0.0125, 0.0250, 0.0375, 0.0500, 0.0625, 0.0750, and 0.0875 s.

The parameters of simulated data are randomly set according to Table S1. Frequency *f* is a random non-repeating number, and the frequency interval between each signal is not less than 4 Hz. It's noted that in order to mimic the overlapping signals, two or three overlapping signals with an interval in [4 Hz, 6 Hz] are randomly added to each simulated spectrum. In addition, to be close to the real situation where experimental data suffer from phase distortion, phase distortion was introduced by multiplying the original FID signal by e^{jr}, where r is in radians, used to control the degree of phase distortion. The range of r in the training set is from -0.3 to 0.3.

The spin echo spectra of estradiol

As an example, 8 spectra of 50 mM estradiol with different echo times are shown in Fig. S5, used as the input of the network.

Processing spectra of α-asarone with additional Gaussian noise

Gaussian random noise with an intensity coefficient of 1.5×10^{-3} was added to the original spin echo phase-changed spectra, as shown in Fig. S6a. It is seen that the weakest signal in the expanded region is impossible to be correctly identified. Through the processing by SE2CSNet, the signal immersed in the strong noise is accurately discerned (Fig. S6d), and the noise is not recognized as the signal.

Variation of chemical shifts of azithromycin with different concentrations

As shown in Fig. S7, with the variation of concentration, the chemical shifts of signal-9" and signal-25 vary, leading to different degrees of overlap of signal-9", signal-4" and signal-23, signal-25 (see expanded regions in Fig. S7). The lower the concentration, the more severe the overlap. It is highly difficult, or impossible to distinguish them at the concentration of 20 mM, even from the PSYCHE spectrum.

Processing simulated spectra with a weak doublet and strong noise

A set of spin echo phase-changed spectra with different levels of noise (different wSNRs) was simulated to test the limit of wSNR. Each spectrum consists of a strong singlet and a weak doublet. As shown in the Fig. S8, when wSNR is greater than 4, the network could accurately and stably discern weak signals from noise, but without recognizing noise as signals even when noise is stronger than the weakest signal.

Processing simulated spectra with different degrees of signal overlapping

In order to test the ability of the network to discern overlapping signals, two sets of simulated spin echo phase-changed spectra were generated. In one set, two doublets overlap with each other with exactly the same transverse relaxation times, *J*-coupling constants, signal intensities, but with slightly different chemical shifts (see the pink box in Fig. S9a). In the other set, a singlet and one of the splitting signals of a doublet are completely overlapped (the chemical shifts of the singlet and the splitting signal are exactly the same) (see the pink box in Fig. S9b). As shown in the Fig. S9, the network model could successfully deal with the two cases, correctly identifying chemical shifts.

Processing simulated spectra with large *J*-coupling constants

A set of spin echo phase-changed spectra with large *J*-coupling constants was simulated to test the network's generalization ability in dealing with heteronuclear coupling. Each set includes a large one-band *J*-coupling constant. The result shows that the network can accommodate *J*-coupling constants up to 24 Hz, as shown in Fig. S10.

Processing simulated spectra with different degrees of strong coupling

A set of spin echo phase-changed spectra with different degrees of strong coupling was simulated to test the network's generalization ability in this regard. The strong coupling signal were generated based on the expression $\{\cos(\pi J_1 t_1) + \alpha j \sin(\pi J_1 t_1)\} \times \{\cos(\pi J_2 t_1) - \alpha j \sin(\pi J_2 t_1)\}$[18], where α is the factor that account for roofing effects (referred to as the strong coupling factor). In order to systematically evaluate SE2CSNet's ability to identify strong coupling signals, α was set as a series of values within the range of 0 to 1. The evaluation results reveal that when the factors are not larger than 0.74, SE2CSNet can correctly identify the signals, as shown in Fig. S11.

Processing simulated spectra with different degrees of phase distortion

A set of spin echo phase-changed spectra with different degrees of phase distortion was simulated to test the network's generalization ability in this regard. Phase distortion can be introduced by multiplying the original FID signal by e^{jr}, where r is in radians, used to control the degree of phase distortion. Fig. S12 shows the test results of the phase distortion threshold, and the critical values tested are -1.59 radians (Fig. S12a) and 1.4 radians (Fig. S12b)

Processing simulated spectra with different line widths

A set of spin echo phase-changed spectra with different line widths was simulated to test the network's generalization ability in this regard. Fig. S13 shows that the test result of the line width threshold and that the maximum line width that the network can accommodate is 22 Hz.

Processing simulated spectra with strong solvent signal

A set of spin echo phase-changed spectra with different intensities of strong solvent signal was simulated to test the network's generalization ability in this regard. Since the input spectra are normalized, the signal height is scaled to a range of 0 to 1 regardless of its original strength. A single peak with a height of 1 can be used to mimic the solvent peak, and the ratio of the lowest signal to the solvent peak (the highest signal) can be used to evaluate SE2CSNet's ability to identify signals in the presence of a strong solvent peak. As shown in the Fig. S14, when the ratio of the lowest signal to the highest peak is not smaller than 0.1%, SE2CSNet can identify the signal.

Processing experimental spectra with bad shimming

When shimming coils are offset, field homogeneity deteriorates, which can result in line shape distortion and increased line width. Fig. S15a shows the experimental spectrum of ibuprofen under optimal shimming conditions, while Figs. S15b and S15c show the spectra of ibuprofen after increasing the value of Z_1 shimming coil by 80 and increasing the value of shimming coil Z_2 by 150, where line widths become broader and the asymmetry increases compared to S15a. The results indicate that under the two conditions, SE2CSNet still retains the ability to correctly identify the signal. However, it is important to note that SE2CSNet was only trained on optimally shimmed simulated spectra, so the assessment of field inhomogeneity represents an application to data that are beyond the range of the training datasets.

Processing experimental spectra with pulse miscalibrations

Miscalibration in the pulse widths (non-optimal pulse flip angles) can lead to signal changes. Fig. S16 shows spin echo experimental spectra of 6 mM ibuprofen collected using pulses with flip angles deviating from optimal ones. Specifically, spectra were acquired using 80-degree and 160-degree pulses (Fig. S16a), and using 140-degree pulses and 280-degree pulses (Fig. S16b) replacing 90-degree and 180-degree pulses. It can be observed that the signal intensities change under the two conditions. It is worth noting that SE2CSNet was trained only on simulated datasets generated with ideal 90-degree and 180-degree pulses, but it can accommodate deviation of flip angles by -10-degree to 50-degree from optimal 90-degree, by -20-degree to 100-degree from optimal 180-degree.

Fig. S1 to 16

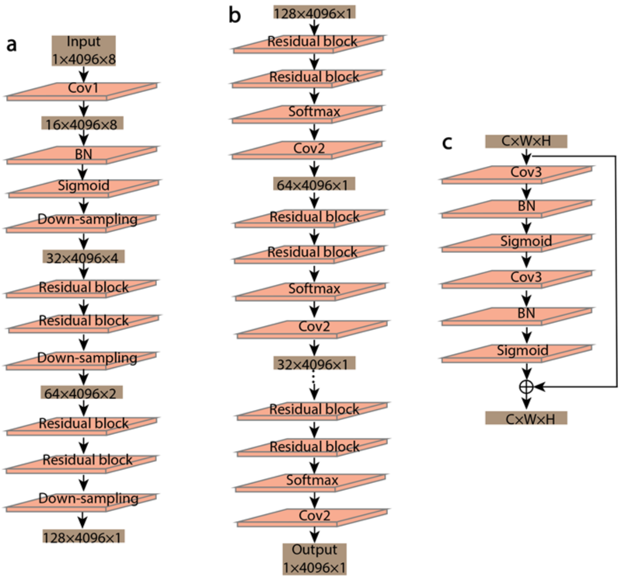

Fig. S1 Details of SE2CSNet. (a) Down-sampling module in the indirect dimension. (b) Dimension-merging module in the channel dimension. 'Down-sampling', 'Conv1' and 'Conv2' are 2D convolutional layers, where the "filter sizes" are (3, 3), (3, 3) and (1, 1) respectively, the "strides" are (1, 2), (1, 1) and (1, 1) respectively, the "padding" is (1, 1), (1, 1) and (0, 0) respectively, and the output channels are respectively 2, 16 and 1/2 times of the input channels. 'Softmax' and 'Sigmoid' are the activation layers. (c) Residual block. 'Conv3' and 'Conv4' are 2D convolutional layers, where the "filter sizes" are (1, 1) and (3, 3) respectively, the "strides" are both (1, 1), and the "padding" is (0, 0) and (1, 1) respectively. 'BN' is the batch normalization layer and 'Sigmoid' is the activation layer.

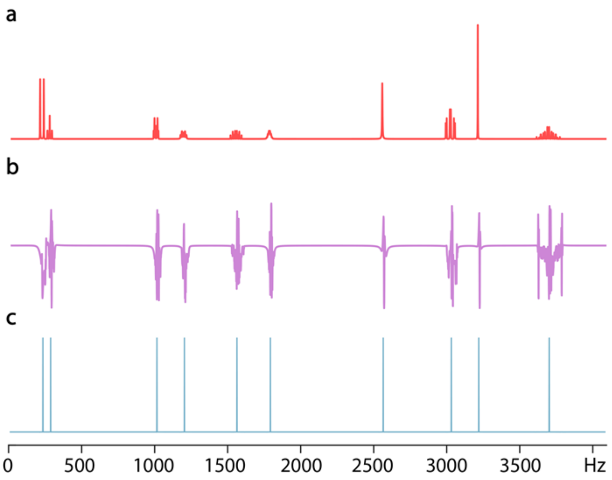

Fig. S2 The visualization of the output of the two modules in SE2CSNet. (a) The spin echo spectrum with an echo time of 0 s. (b) One channel of the output feature vector from the down-sampling module in the indirect dimension. (c) The output from the dimension-merging module in the channel dimension.

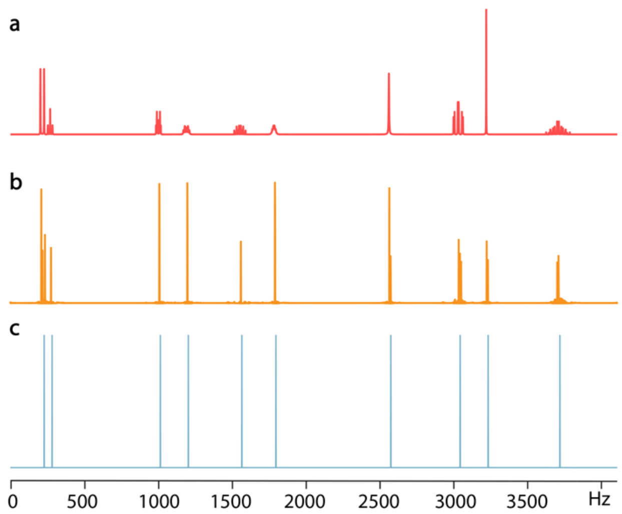

Fig. S3 The comparison between two different ways of dimension-merging (a) The spin echo spectrum with an echo time of 0 s. (b) Spectrum with the way of in-one-step. (c) Spectrum with the way of step-by-step.

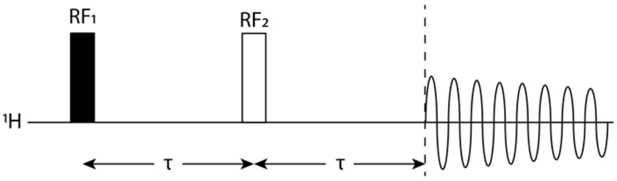

Fig. S4 The spin echo pulse sequence. 'RF1' is a hard π/2 pulse, 'RF2' is a hard π pulse, and 2τ is the echo time.

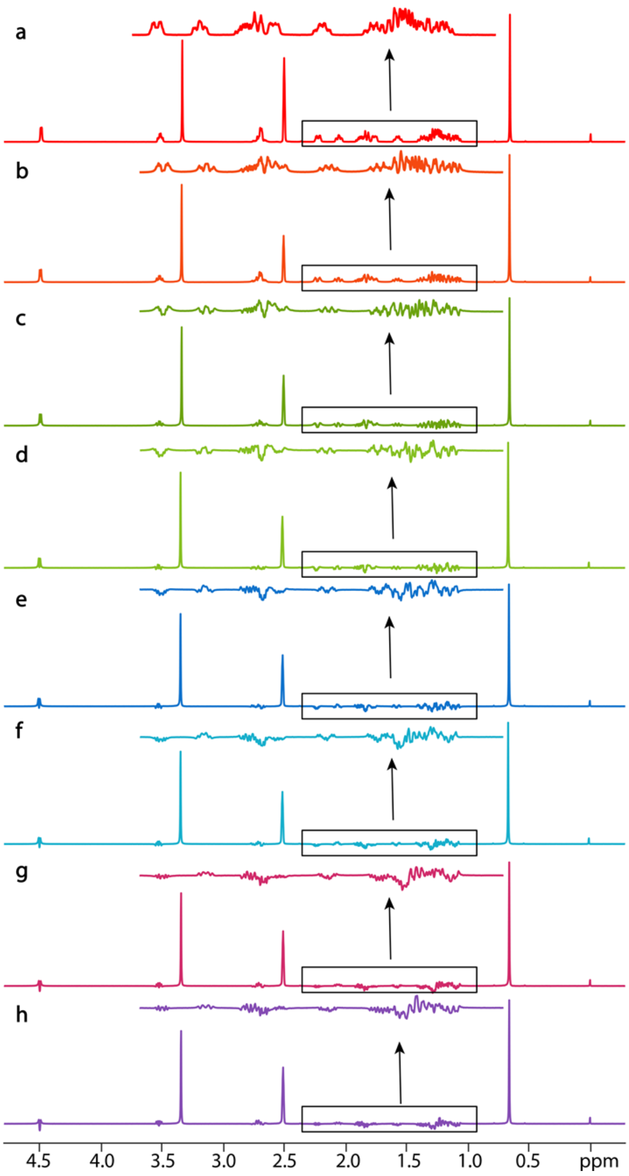

Fig. S5 The spin echo spectra of 50 mM estradiol. The echo times from (a) to (h) are 0, 0.0125, 0.0250, 0.0375, 0.0500, 0.0625, 0.0750, and 0.0875 s, respectively. Representative regions are expanded.

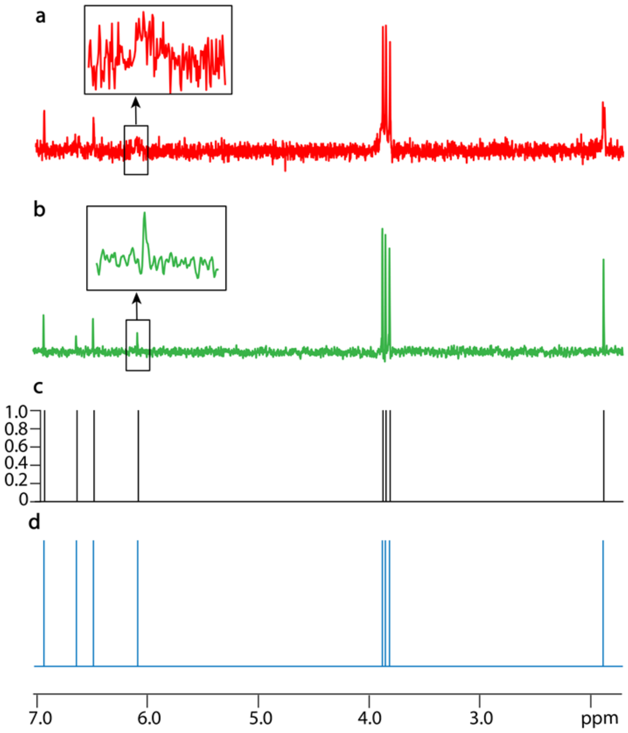

Fig. S6 1D NMR spectra of α-asarone of 1 (a and c) and 30 mM (b) dissolved in Chloroform-d (CDCl3) with a scan number of one. (a) The spin echo spectrum with an echo time of 0 s with additional random noise. (b) The PSYCHE spectrum. (c) The confidence result produced by SE2CSNet. (d) The output chemical shift spectra produced by SE2CSNet.

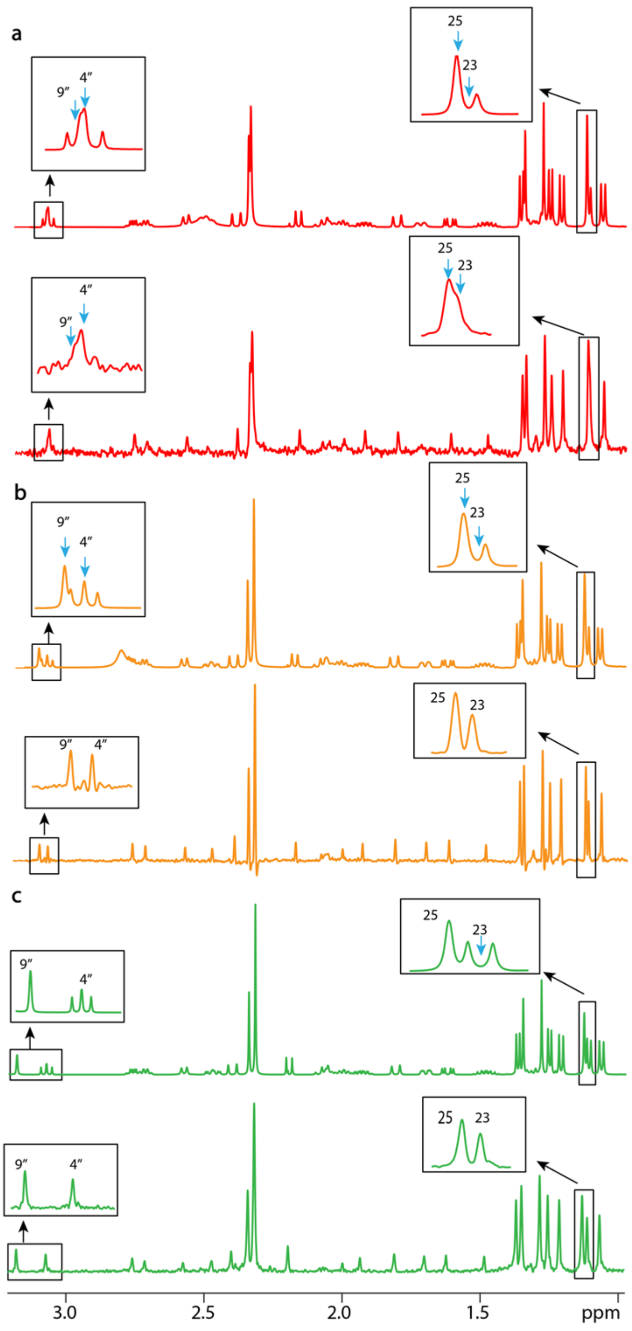

Fig. S7 Spectra of azithromycin at different concentrations. (a) - (c) are the spin echo spectrum with an echo time of 0 s and the PSYCHE spectrum at concentrations of 20 (a), 40 (b) and 100 (c) mM, respectively. Representative regions are expanded. Blue arrows indicate representative signals. Blue arrows point to chemical shift values.

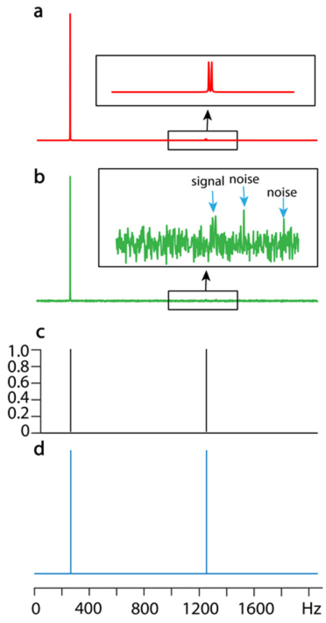

Fig. S8 Test of SE2CSNet on one simulated dataset with a weak doublet. (a) The conventional spectrum without noise. (b) The input spectrum with wSNR of 4. (c) The confidence result produced by SE2CSNet. (d) The output chemical shift spectra produced by SE2CSNet.

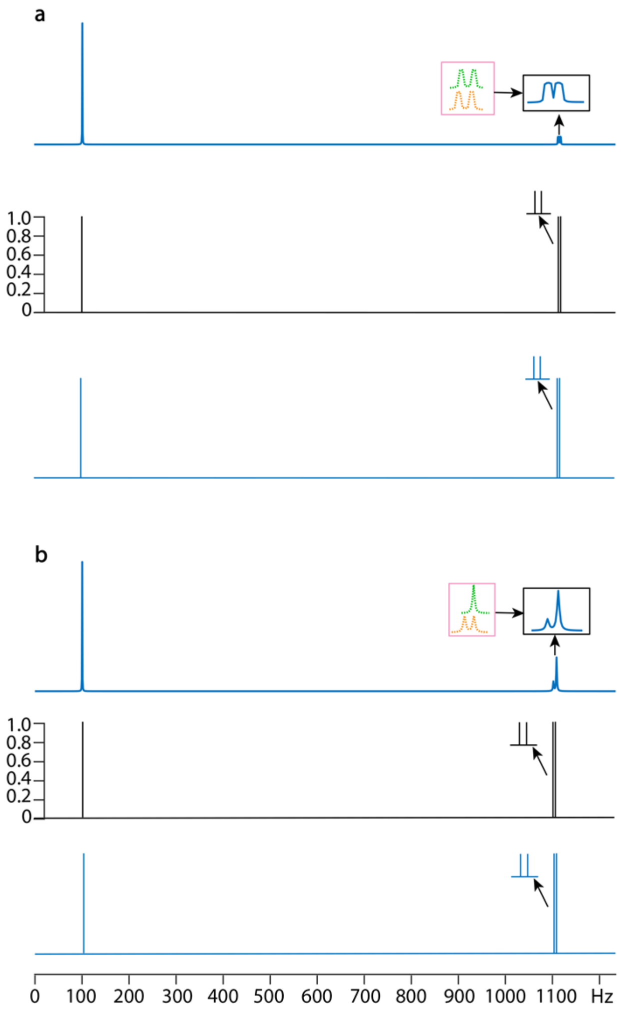

Fig. S9 Testing on the simulated datasets with severe overlapping. (a) From top to bottom: the simulated input spectra with the overlapping of two doublets, the confidence result and the output chemical shift spectra produced by SE2CSNet. (b) From top to bottom: the simulated input spectra with the overlapping of a singlet and a doublet, the confidence result and the output chemical shift spectra produced by SE2CSNet.

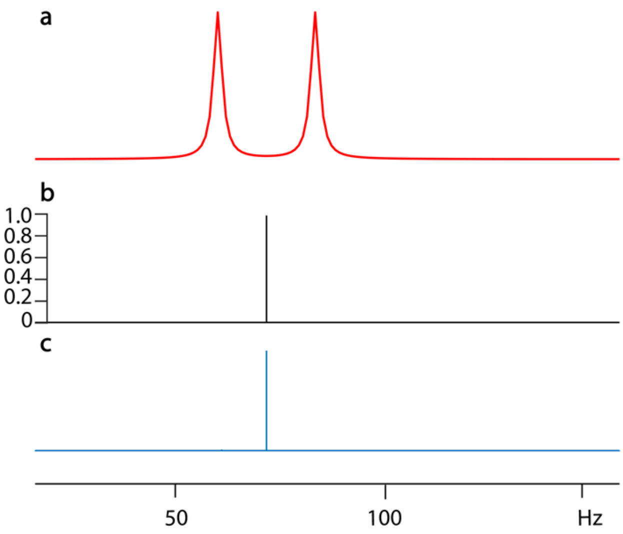

Fig. S10 Assessing the performance of SE2CSNet on large one-band *J*-coupling constants. (a) The simulated input spectrum with the *J*-coupling constant set to 24 Hz. (b) The confidence result produced by SE2CSNet. (c) The output chemical shift spectrum produced by SE2CSNet.

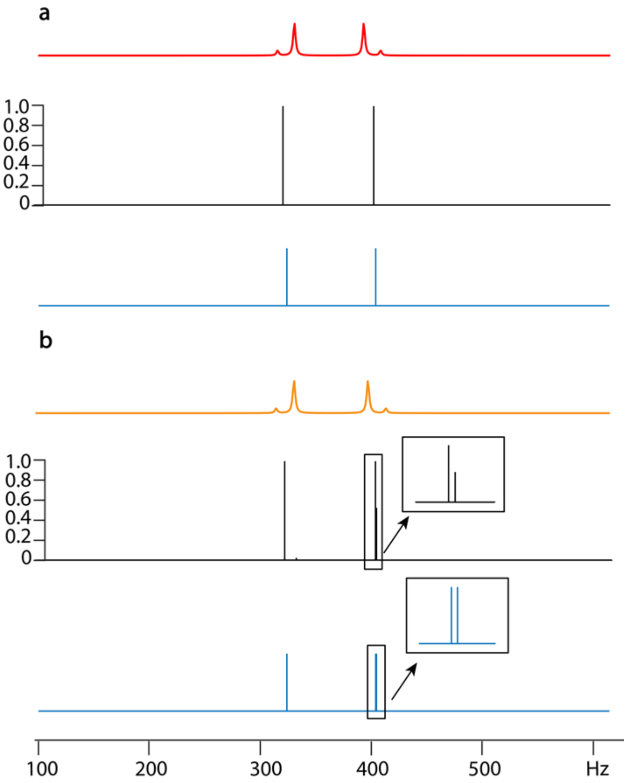

Fig. S11 Assessing the performance of SE2CSNet on identifying strong coupling signals. (a) From top to bottom: the simulated input spectra with the strong coupling factor α set to 0.74, the confidence result and the output chemical shift spectra produced by SE2CSNet. (b) Counterpart of (c) with α set to 0.75.

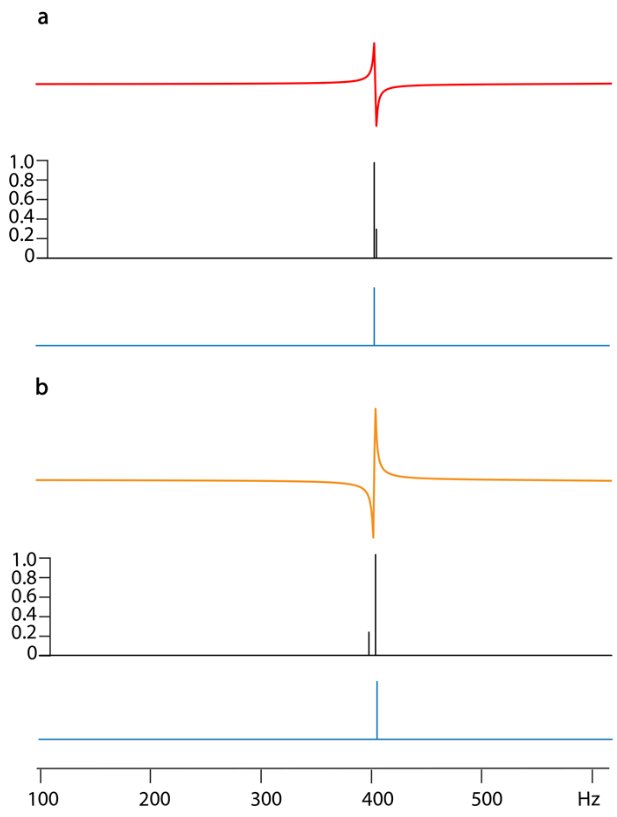

Fig. S12 Assessing the performance of SE2CSNet on phase distortion. (a) From top to bottom: the simulated input spectra with the phase set to -1.59 radians, the confidence result and the output chemical shift spectra produced by SE2CSNet. (b) Counterpart of (a) with the phase set to 1.4 radians.

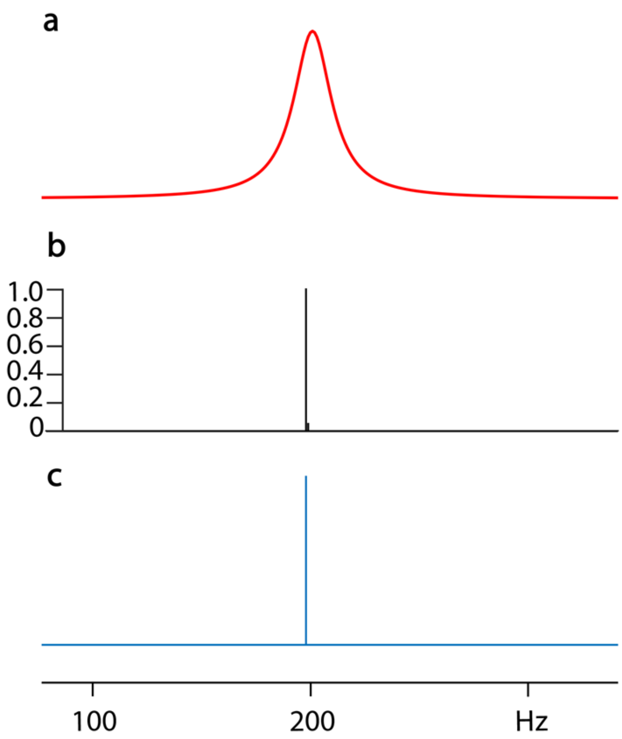

Fig. S13 Assessing the performance of SE2CSNet on spectra with large line widths. (a) The simulated input spectrum with the line width set to 22 Hz. (b) The confidence result produced by SE2CSNet. (c) The output chemical shift spectrum produced by SE2CSNet.

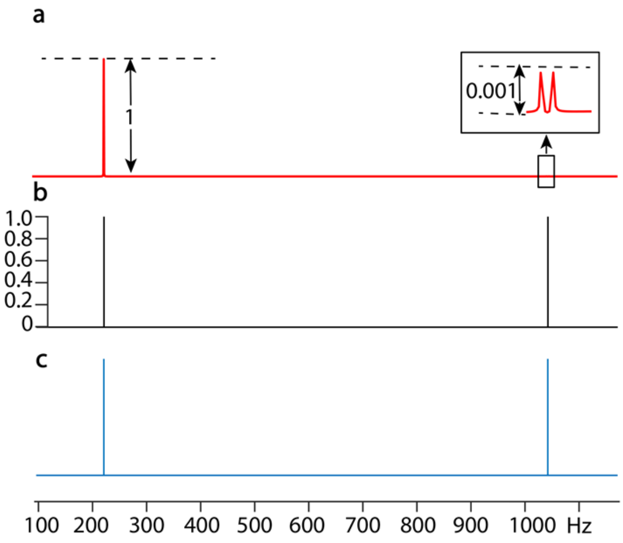

Fig. S14 Assessing the performance of SE2CSNet on a spectrum with a strong solvent signal. (a) The simulated input spectrum with the ratio of the lowest signal to the highest peak set to 0.1%. (b) The confidence result produced by SE2CSNet. (c) The output chemical shift spectra produced by SE2CSNet.

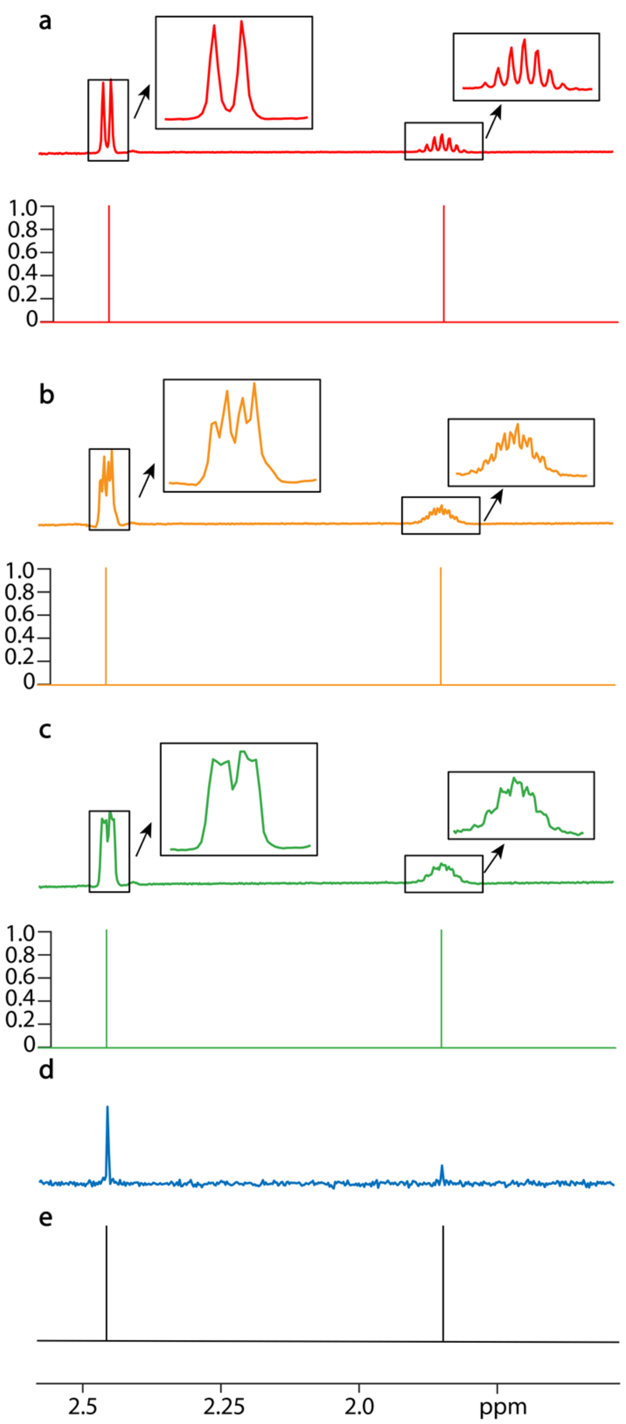

Fig. S15 Assessing the performance of SE2CSNet on accommodating bad shimming. Spectra of 6 mM ibuprofen dissolved in (methyl sulfoxide)-d6 (DMSO-d6) with a scan number of one were acquired at a temperature of 298 K on a 500 MHz Varian NMR spectrometer. (a) The spin echo spectrum with an echo time of 0 s and with optimal shimming, and its confidence result produced by SE2CSNet. (b) The spin echo spectrum with an echo time of 0 s and with the increased value of Z_1 shimming coil by 80 relative to (a), and its confidence result produced by SE2CSNet. (c) The spin echo spectrum with an echo time of 0 s and with the increased value of Z_2 shimming coil by 150 relative to (a), and its confidence result produced by SE2CSNet. (d) The PSYCHE spectrum as a reference. (e) The output chemical shift spectra of (a, b and c) produced by SE2CSNet.

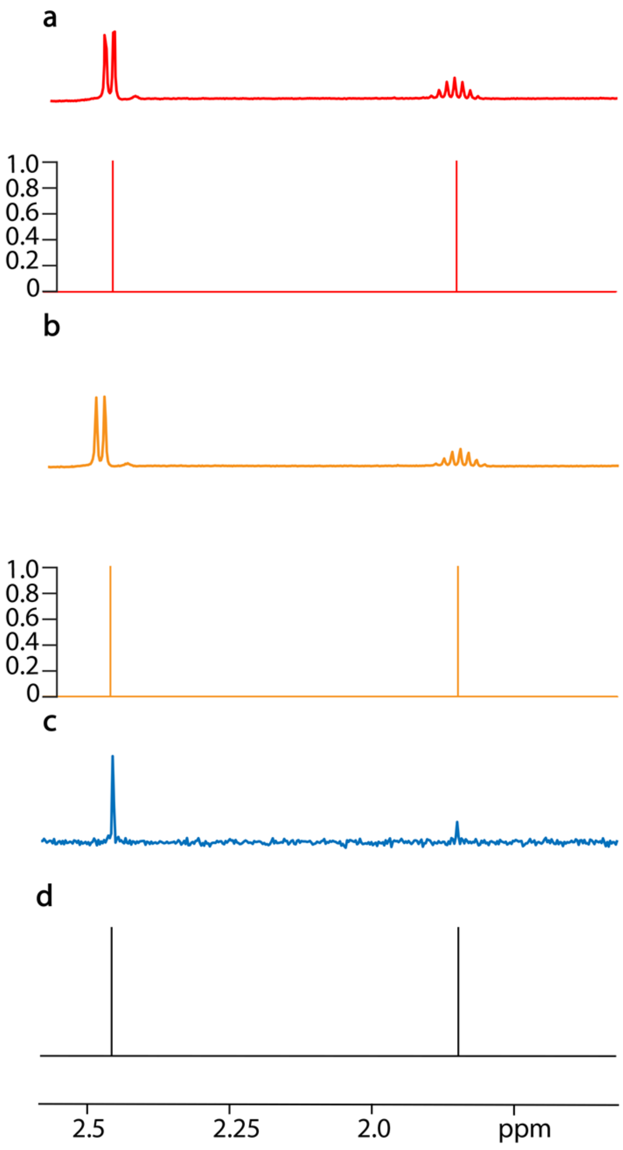

Fig. S16 Assessing the performance of SE2CSNet on data collected with non-optimal flip angles. Spectra of 6 mM ibuprofen dissolved in (methyl sulfoxide)-d6 (DMSO-d6) with a scan number of one were acquired at a temperature of 298 K on a 500 MHz Varian NMR spectrometer. (a) The spin echo spectrum collected with 80-degree pulses and 160-degree pulses replacing 90-degree and 180-degree pulses, and its confidence result produced by SE2CSNet. (b) The spin echo spectrum collected with 140-degree pulses and 280-degree pulses replacing 90-degree and 180-degree pulses, and its confidence result produced by SE2CSNet. (c) The PSYCHE spectrum as a reference. (e) The output chemical shift spectra of (a and b) produced by SE2CSNet.

Table S1

Table S1 Parameter ranges of simulated NMR FID signal.

Parameter	N	A	f (Hz)	T_2 (s)	J (Hz)	pow
Minimum	15	1	10	0.15	0	0
Maximum	45	50	SW-10	1	15	3
